# Role of MEK/ERK pathway in the MAD2-mediated cisplatin sensitivity in testicular germ cell tumour cells

**DOI:** 10.1038/sj.bjc.6603284

**Published:** 2006-08-01

**Authors:** M K L Fung, H-W Cheung, M-T Ling, A L M Cheung, Y-C Wong, X Wang

**Affiliations:** 1 Cancer Biology Group, Department of Anatomy, Laboratory Block, Faculty of Medicine, The University of Hong Kong, 21 Sassoon Road, Hong Kong, SAR, China

**Keywords:** MAD2, MEK, cisplatin, TGCT

## Abstract

Testicular germ cell tumour (TGCT) is the most common malignancy in young males. Although most TGCTs are sensitive to cisplatin-based chemotherapy, significant numbers of TGCT patients still relapse and die each year because of the development of resistance to cisplatin. Previously, we first reported that a key regulator of the mitotic checkpoint, mitotic arrest deficient-2 (MAD2), was a mediator of cisplatin sensitivity in human cancer cells. In this study, we investigated whether MAD2 played a role in cellular sensitivity to cisplatin in TGCT cells and the underlying molecular mechanisms responsible. Using 10 TGCT cell lines, we found that increased MAD2 expression was correlated with cellular sensitivity to cisplatin, which was associated with activation of the MEK pathway. Treatment of cells expressing high levels of MAD2 with an MEK inhibitor, U0126, led to cellular protection against cisplatin-induced apoptosis. Inactivation of MAD2 by transfecting a dominant-negative construct in TGCT cells with high levels of MAD2 resulted in the suppression of MEK pathway and resistance to cisplatin-induced cell death. These results support previous suggestion on the involvement of mitotic checkpoint in DNA damage response in human cancer cells and demonstrate a possible molecular mechanism responsible for the MAD2-mediated sensitivity to cisplatin in TGCT cells. Our results also suggest that downregulation of MAD2 may be an indicator for identification of TGCT cancer cells that are potentially resistant to cisplatin-based therapy.

Testicular germ cell tumour (TGCT) is the most common type of malignancy in young males aged 15–35 years and the incidence is increasing steadily throughout the world (McGlynn *et al*, 2003). In the past 30 years, the incidence of TGCT has doubled, especially in young men with a mean age of 36 years at diagnosis (Power *et al*, 2001). Since the introduction of cisplatin-based chemotherapy in the mid-1970s, the mortality of this disease has been reduced significantly and the 5-year survival rate can be achieved in over 85% of patients when detected early (Jones and Vasey, 2003). Hence, cisplatin is considered as the most efficient single agent in the treatment of TGCT. However, owing to the development of cisplatin resistance, a number of young patients die every year.

Multiple mechanisms have been suggested in the development of cisplatin resistance including reduction of intracellular drug accumulation, overexpression of thiol-containing molecules, increased DNA damage repair, upregulation of antiapoptotic genes as well as defects in certain signal-transduction pathways (Siddik, 2003). Recently, mitogen-activated protein kinase (MAPK) (Lavoie *et al*, 1996) signalling pathway has been suggested to play a role in cisplatin-mediated cytotoxicity in carcinoma cells (Siddik, 2003). There are three major MAPK subfamilies: extracellular signal-regulated kinases (ERK), c-jun N-terminal kinases and p38 kinases. These MAPK members participate in integrating extracellular signals to regulate cell proliferation, differentiation, cell survival and apoptosis (Dent and Grant, 2001). Wang *et al* (2000) have shown that all three kinase members are activated after exposure to cisplatin in cervical cancer cells. They, however, suggest that only ERK activation is the most important factor for cisplatin-induced apoptosis, which is consistent with the demonstration that the cisplatin-induced ERK activation contributes to regulation of p53 by phosphorylating the tumour suppressor at serine-15 (Persons *et al*, 2000). Moreover, Yeh *et al* (2002) have also reported that inhibition of the MEK–ERK pathway leads to cisplatin resistance in cervical carcinoma cells. Contradictory results have also been reported that activation of ERK pathway by cisplatin antagonises apoptosis in ovarian carcinoma cell lines (Persons *et al*, 1999). In addition, inhibition of cisplatin-induced ERK activation enhances sensitivity to cisplatin in both cisplatin-sensitive and cisplatin-resistance ovarian cells carcinoma cell lines (Cui *et al*, 2000). These studies indicate the importance of the MEK/ERK pathway in cisplatin-induced apoptosis. Recently, a study has shown that MEK/ERK activation is also important in cisplatin-induced apoptosis in human TGCT cell lines. The authors have shown that after cisplatin treatment, MEK and ERK are dually phosphorylated which is associated with caspase 3 activation. Treatment with MEK inhibitors not only downregulates the level of ERK phosphorylation but also reduces the apoptotic rate of cisplatin-treated TGCT cell lines (Schweyer *et al*, 2004a). These results suggest that activation of MEK/ERK pathway may play an important role in cisplatin-induced cell death in TGCT cells.

Recently, we have demonstrated that mitotic arrest deficient-2 (MAD2) plays a key role in cisplatin sensitivity in nasopharyngeal carcinoma (NPC) cells (Cheung *et al*, 2005). Mitotic arrest deficient-2 is one of the key regulators of the mitotic checkpoint, which plays an essential role in the proper segregation of chromosomes in each cell division (Rudner and Murray, 1996). Recently, the MAD2-mediated mitotic checkpoint has been shown to be involved in cellular response to DNA damage in both human and yeast cells (Garber and Rine, 2002; Mikhailov *et al*, 2002). Previously, we have found that ectopic MAD2 expression in NPC cells leads to increased sensitivity to cisplatin via activation of the apoptosis pathway (Cheung *et al*, 2005). In addition, we have shown that the MAD2-mediated chemosensitivity to vincristine, a microtubule-disrupting agent, is associated with activation of the MEK pathway (Wang *et al*, 2003). These results suggest that MAPK pathways may be important in MAD2-mediated sensitivity to certain anticancer drugs.

The aim of this study was to investigate whether MAD2 played a role in cisplatin-induced apoptosis in TGCT cells and whether MEK/ERK pathway was involved in this process. Using 10 TGCT cell lines, we found that increased MAD2 expression was correlated with sensitivity to cisplatin and activation of the MEK/ERK pathway. Suppression of MEK/ERK pathway in cells with high levels of MAD2 conferred resistance to cisplatin. Inactivation of MAD2, on the other hand, led to suppression of the MEK/ERK pathway and decreased sensitivity to cisplatin-induced apoptosis. Our results suggest that the MAD2-mediated sensitivity to cisplatin may be regulated through modification of the MEK pathway.

## MATERIALS AND METHODS

### Cell lines and cell culture conditions

Ten TGCT cell lines were used in this study. Six TGCT cell lines were (GH, 833K, SuSa, SuSa-CP, GCT27 and GCT27R) kindly provided by Professor J Masters (University College, London, UK) and a human cervical carcinoma cell line, HeLa, obtained from American Type Culture Collection (Rockville, MD, USA) was used as a positive control. They were maintained in RPMI 1640 (Invitrogen, Carlsbad, CA, USA) supplemented with 5% fetal bovine serum (FBS) and penicillin/streptomycin. Three TGCT cell lines (1411HP, NT2/D1, 2102EP) obtained from Professor P Andrews (University of Sheffield, UK) were grown in DMEM (Invitrogen, Carlsbad, CA, USA) supplemented with 10% FBS and penicillin/streptomycin, and NCCIT cell line (kindly provided by Professor C Bokemeyer; University of Tuebingen, Germany) was cultured in RPMI 1640 supplemented with 10% FCS and penicillin/streptomycin. All cell lines were cultured at 37°C in a humidified atmosphere with 5% CO_2_. The characteristics and the origin of all the TGCT cell lines used in this study are listed in Table 1. The MEK1/2 inhibitor, U0126, was purchased from Cell Signaling Technology (Beverly, MA, USA).

### Colony-forming assay

One thousand to 3000 cells were plated in 12-well plates according to the individual plating efficiencies. Cisplatin (David Bull Laboratories, Victoria, Australia) or Taxol (Calbiochem, San Diego, CA, USA) was added 24 h after plating. After 10–14 days of incubation, the cells were fixed in 70% ethanol and stained in 10% Giemsa. Colonies consisting of 50 or more cells were counted and survival curves were plotted. Two wells were used for each concentration and the corresponding controls. Each experiment was repeated at least three times and each data point represented the mean and standard derivation.

### Western blotting

Cell lysates were prepared by suspending cell pellets in lysis buffer (50 mmol l^−1^ Tris-HCl (pH 8.0), 150 mmol l^−1^ NaCl, 1% NP40, 0.5% sodium deoxycholate and 0.1% SDS) containing proteinase inhibitors (1 *μ*g ml^−1^ aprotinin, 1 *μ*g ml^−1^ leupeptin and 1 mmol l^−1^ phenylmethylsulphonyl fluoride). Protein concentration was measured using DC Protein Assay kit (Bio-Rad, Hercules, CA, USA). Same amount of protein (30 *μ*g) was loaded onto a SDS–polyacrylamide gel for electrophoresis and then blotted onto a nitrocellulose membrane (Amersham, Piscataway, NJ, USA). After blocking with 10% nonfat milk or 5% bovine serum albumin in TBS-T for 1 h, the membrane was incubated with primary antibodies for 1 h at room temperature against MAD2 (BD Transduction Laboratories, Franklin Lakes, NJ, USA), p-MEK, p-p44/42 MAP kinase, p-Elk-1, PARP (Cell Signaling Technology, Beverly, MA, USA), HA (Roche Diagnostics, Indianapolis, IN, USA) or actin (Santa Cruz Biotechnology, Santa Cruz, CA, USA). Then, the membrane was incubated with suitable secondary antibodies against rabbit immunoglobulin G, mouse immunoglobulin G or goat immunoglobulin G (Amersham, Piscataway, NJ, USA). Signals were visualised by enhanced chemiluminescence Western blotting system (Amersham, Piscataway, NJ, USA). Expression of actin was also used as an internal loading control.

### Immunocytochemistry

Five thousand cells were plated on chamber slides of 28 mm^2^ overnight. The cells were fixed in 4% paraformaldehyde in PBS for 25 min. The cells were immersed in 0.6% H_2_O_2_ for 30 min. Immunocytochemistry was then performed with the use of Vectastain® Elite ABC Kit (ABC kit) according to the manufacturer's manual (Vector Laboratories Inc., Burlingame, CA, USA). Briefly, nonspecific binding of the antibodies was blocked by 10% normal horse serum provided in the ABC kit for 20 min. Next, the slides were incubated with MAD2 antibodies (BD Transduction Laboratories, Franklin Lakes, NJ, USA) in 10% normal horse serum at 4°C overnight. They were then incubated with biotinylated secondary antibodies provided in the ABC kit at room temperature for an hour. They were then incubated with ABC reagent at room temperature for 30 min. Signals were visualised with diaminobenzidine (Dako Corp., Hamburg, Germany). Cells were counterstained with Mayer's haematoxylin and were mounted. MAD2-positive expression was stained as brown signals in both nucleus and cytoplasm. The overall staining intensity (both nuclear and cytoplasmic staining) was scored using a grading scale as 0 (negative), 1 (weak), 2 (moderate) or 3 (strong).

### Terminal deoxynucleotidyl transferase-mediated nick end labelling

Five thousand cells were plated onto chamber slides of 28 mm^2^. Cisplatin was added 24 h after plating and incubated for 4 days. The cells were fixed in 4% paraformaldehyde in PBS for 25 min. *In situ* death detection kit, Fluorescein, was used to detect apoptotic cells according to the manufacturer's manual (Roche Diagnostics, Indianapolis, IN, USA). The percentage of terminal deoxynucleotidyl transferase-mediated nick-end labelling (TUNEL)-positive cells was calculated as the number of TUNEL-positive cells over the total number of cells counted × 100. At least 500 cells were counted from three random fields under × 200 magnification in each experiment. Each experiment was repeated twice. Each data point represented the mean and standard derivation. *P*<0.05 was considered statistically significant as determined by two-tailed Student's *t*-test.

### 3-(4,5-dimethyl thiazol-2-yl)-2,5-diphenyl tetrazolium bromide assay

3-(4,5-Dimethyl thiazol-2-yl)-2,5-diphenyl tetrazolium bromide assay proliferation assay was performed according to the manufacturer's manual (Roche Diagnostics, Indianapolis, IN, USA). Briefly, 3000 cells were plated in 96-well plates and cisplatin was added 24 h later for indicated time points. Following overnight culture, 10 *μ*l of MTT labelling reagent was added and the cells were incubated for 4 h at 37°C. Afterwards, 100 *μ*l of solubilising reagent was added and the plates were incubated at 37°C overnight. Absorbance at 570 nm was measured with Labsystem multiscan microplate reader (Merck Eurolab, Dietikon Schweiz). Each time point was carried out in triplicate and each experiment was repeated at least twice. Each data point represented the mean and standard deviation. *P*<0.05 was considered statistically significant as determined by two-tailed Student's *t*-test.

### Transfection

GH cells were seeded 24 h before transfection and semiconfluent monolayer cells were transfected with a HA-tagged dominant-negative construct of MAD2 (MAD2ΔC) using Fugene 6 Transfection Reagent (Roche Diagnostics, Indianapolis, IN, USA) according to the manufacturer's manual. For plasmid construction, a 1.5-kb fragment containing HA-tagged truncated form of human MAD2 cDNA (MAD2ΔC) was cloned into pLenti6/V5-D-TOPO expression vector by TOPO cloning reaction (Invitrogen, Carlsbad, CA, USA). MAD2ΔC lacks the C-terminal 10 amino acids that is necessary for the function of MAD2 (Canman *et al*, 2002). Cisplatin was added 48 h post-transfection. The transfected cells were treated and harvested at indicated time points for further analysis. For the control experiments, cells were transfected with pLenti6/V5-D-TOPO expression vector alone.

## RESULTS

### Increased MAD2 expression correlates with sensitivity to cisplatin

**Figure 1:**
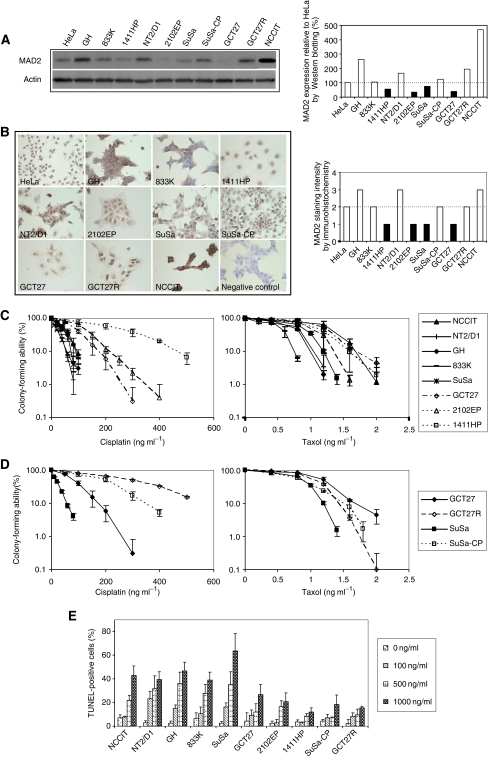
Correlation of MAD2 expression and sensitivity to cisplatin and taxol. (**A**) MAD2 expression in 10 TGCT cell lines GH, 833K, 1411HP, NT2/D1, 2102EP, SuSa, SuSa-CP, GCT27, GCT27R and NCCIT analysed by Western blotting. A human cervical carcinoma cell line HeLa was used as a positive control. (**B**) Representative results of immunohistochemical staining of MAD2 in HeLa, GH, 833K, 1411HP, NT2/D1, 2102EP, SuSa, SuSa-CP, GCT27, GCT27R, NCCIT cells and a negative control with primary antibody omitted. Photographs were taken under × 400 magnifications. (**C**) Colony-forming ability of TGCT cell lines with high (solid lines) and low (dotted lines) levels of MAD2 protein after exposure to cisplatin (left panel) and taxol (right panel). Note that cells with high levels of MAD2 (solid lines) were more sensitive to cisplatin but not to taxol. Results represented means of three independent experiments and error bars indicated standard deviation. (**D**) Colony-forming assay of acquired cisplatin-resistant cell lines and their parental lines. Results represented means of three independent experiments and error bars indicated standard deviation. (**E**) Terminal deoxynucleotidyl transferase-mediated nick end labelling staining of the 10 TGCT cell lines after treatment with three doses of cisplatin for 4 days. Note that the percentage of TUNEL-positive cells was higher in cell lines with high level of MAD2 (NCCIT, NT2/D1, GH, 833K and SuSa) than cell lines with low levels of MAD2 (GCT27, 2102EP and 1411HP). Results represented means of three independent experiments and error bars indicated standard deviation.

To investigate whether MAD2 expression was associated with cellular sensitivity to cisplatin, we first examined the expression of MAD2 in 10 TGCT cell lines using HeLa cell line as a positive control because it was shown to express a relatively high level of MAD2 (Wang *et al*, 2000). As shown in Figure 1A, 40% of the TGCT cell lines (four out of 10) expressed lower level of MAD2 compared to HeLa cells. Among the 10 TGCT cell lines tested, MAD2 protein level was the lowest in GCT27, 2102EP and 1411HP cell lines and it was the highest in NCCIT and GH cell lines (Figure 1A). To confirm these results, we performed immunohistochemical staining of MAD2 on the cell lines, and we observed that MAD2 expression was found in both nucleus and cytoplasm. The overall MAD2 expression intensity in 1411HP, 2102EP and GCT27 cell lines was weak compared to NCCIT and GH cell lines (Figure 1B). These results supported our results generated from Western blotting.

Colony-forming assay on the cellular sensitivity to cisplatin showed that the three cell lines with low MAD2 protein expression (dotted lines) were more resistant than the ones with relatively high MAD2 expression (solid lines) (Figure 1C). For the convenience of comparison, we calculated the inhibition concentrations of 50 and 90% colony-forming ability (IC_50_, IC_90_) shown in Table 2. IC_50_ and IC_90_ concentrations required for the cells with low MAD2 expression were much higher than the cell lines with high MAD2 expression (up to six-fold). These results indicated that attenuated MAD2 expression in TGCT cells was associated with decreased sensitivity to cisplatin (*P*<0.01). This association between MAD2 and cisplatin sensitivity was consistent with our previous studies showing that NPC cell lines with low MAD2 protein expression were more resistant to cisplatin-induced apoptosis than cells with relatively high levels of MAD2 expression (Cheung *et al*, 2005). However, a reverse correlation of MAD2 expression and sensitivity to cisplatin was found in acquired cisplatin-resistant pairs (SuSa, SuSa-CP and GCT27, GCT27R) (Figure 1D), suggesting differential roles of MAD2 in intrinsic and acquired cisplatin resistance. To confirm these results, we performed TUNEL assay to study if the differential sensitivity to cisplatin in these cell lines was correlated to apoptosis rate. As shown in Figure 1E, we found that in agreement with the results generated from colony-forming assay, the cell lines expressing relatively high levels of MAD2 showed much higher percentage of TUNEL-positive cells compared to the cells with low levels of MAD2 after treatment with same doses of cisplatin (*P*<0.05).

In order to test whether the MAD2-induced chemosensitivity also occurred to another anticancer drug, taxol, a microtubule stabilising agent, we performed colony-forming assay after treatment with five doses of taxol on all of the cell lines. We found that unlike observed in the cells treated with cisplatin, the association of MAD2 and chemosensitivity to taxol was much less significant (Figure 1C and D, right panels). These findings also agreed with our previous observation that the expression of MAD2 had no significant effect on the chemosensitivity to taxol in NPC cells (Wang *et al*, 2003).

### Suppression of MEK1/2, ERK1/2 and Elk-1 phosphorylation is associated with cisplatin resistance in cells with low level of MAD2

**Figure 2:**
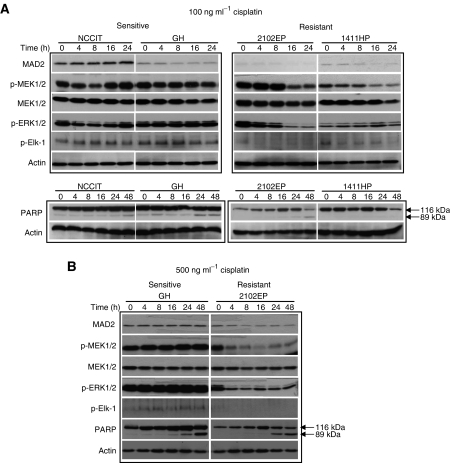
Effect of cisplatin on the expression of MEK pathway-related proteins and PARP. Two TGCT cell lines expressing relatively high (NCCIT, GH) and low levels of MAD2 (2102EP and 1411HP) were examined by Western blotting. Expression of MEK pathway-related proteins and PARP was analysed after treatment with 100 ng ml^−1^ (**A**) and 500 ng ml^−1^ (**B**) of cisplatin. Note that the phosphorylation of MEK pathway-related proteins as well as cleaved PARP were suppressed in relatively resistant cells compared to the sensitive lines.

As discussed previously, activation of MEK/ERK pathway is critical for cisplatin-induced apoptosis (Wang *et al*, 2000), and inhibition of MEK/ERK pathway has been suggested to be responsible for cisplatin resistance (Yeh *et al*, 2002). To study whether the MEK/ERK pathway played a role in the MAD2-mediated sensitivity to cisplatin in TGCT cells, we first examined and compared the expression of MEK pathway-related proteins in two cisplatin-sensitive cell lines with relatively high levels of MAD2 (NCCIT and GH) and two cisplatin-resistant cell lines with low levels of MAD2 (2102EP and 1411HP) after treatment with cisplatin. As shown in Figure 2A, after treatment with cisplatin (100 ng ml^−1^), the sensitive NCCIT and GH cells had relatively high or sustained levels of p-MEK 1/2, p-ERK1/2 and p-Elk-1. However, in the relatively resistant cells, the levels of phosphorylated MAP kinases were downregulated in a time-dependent manner. As phosphorylation is an indication of activation of these proteins (Wang *et al*, 2000), these results suggest that decreased MAD2 expression is associated with suppression of MAPK pathway in response to cisplatin treatment. To investigate whether the expression of these proteins was related to cisplatin-induced activation of the apoptosis pathway, expression of cleaved PARP was examined by Western blotting. As indicated in Figure 2A, the high levels of p-MEK1/2, p-ERK1/2 and p-Elk-1 in the sensitive cells were correlated with increased cleavage of PARP (89 kDa in size), an indicator of cells undergoing apoptosis (Ame *et al*, 2004), especially at later time points. In contrast, the decreased levels of phosphorylated MEK pathway-related proteins in resistance cells were associated with much lower levels of PARP cleavage in response to cisplatin treatment (100 ng ml^−1^) (Figure 2A). These results were also confirmed in GH and 2102EP cells treated with a higher dose of cisplatin (500 ng ml^−1^) (Figure 2B). These results revealed that inhibition of MEK/ERK/Elk pathway was associated with suppression of cisplatin-induced apoptosis in TGCT cells with low MAD2 expression.

### Inactivation of MEK/ERK pathway by an MEK1/2 inhibitor, U0126, leads to cellular protection against cisplatin

**Figure 3:**
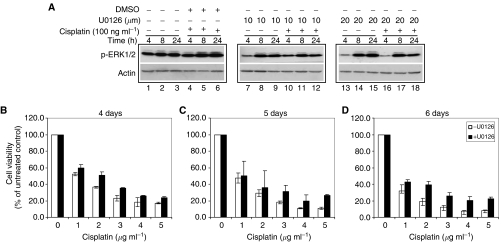
Effect of MEK1/2 inactivation on cisplatin sensitivity in GH cells. GH cells were treated with two doses (10 and 20 *μ*M) of an MEK1/2 inhibitor, U0126, for indicated time points. Western blotting and MTT assay were performed. (**A**) Western blotting analysis of p-ERK1/2 expression before (lanes 1–3) and after exposure to U0126 alone (lanes 7–9; 13–15) and in combination with cisplatin (lanes 10–12; 16–18). The cisplatin- and solvent-treated cells (lanes 4–6) were also tested as an internal control. Note that the expression of p-ERK was lower after exposure to both concentrations of U0126 compared with solvent control in response to cisplatin. (**B–D**) Cell viability after exposure to five concentrations of cisplatin for 4 days (**B**), 5 days (**C**) and 6 days (**D**) in the presence of 20 *μ*M U0126 (filled columns) and absence of U0126 (open columns). Note that after exposure to both cisplatin and U0126, cell viability was higher than that treated with cisplatin alone. Results represented means of three independent experiments and error bars indicated standard deviation.

To further confirm the importance of MEK pathway in MAD2-mediated sensitivity to cisplatin, we next studied whether inhibition of MEK1/2 had any effect on chemosensitivity to cisplatin in TGCT cells with high levels of MAD2. GH cells were chosen because of their relatively high levels of MAD2 and p-MEK1/2, p-ERK1/2 and p-Elk-1 proteins in response to cisplatin. U0126, which had been shown to inactivate MEK-1 and MEK-2 by reducing phosphorylation (Favata *et al*, 1998), was used to evaluate whether inactivation of MEK pathway had any effect on cellular sensitivity to cisplatin. As shown in Figure 3A, after exposure to two concentrations of U0126 (10 and 20 *μ*M), decreased levels of p-ERK1/2 were observed at 4 h (lanes 7 and 13) compared with the untreated control (lane 1). We also noticed that there was a slight increase at later time points (lanes 8, 9, 14 and 15; Figure 3A) which might be due to the gradual recovery of the cells from the treatment or a time-dependent cisplatin-induced MEK/ERK activation. Nevertheless, in comparison with the cells treated with both DMSO (vehicle of U0126) and cisplatin (lanes 4, 5 and 6; Figure 3A), U0126 treatment led to suppression of cisplatin-induced p-ERK1/2 in GH cells (lanes 10, 11, 12, 16, 17 and 18; Figure 3A). After exposure to both U0126 (10 and 20 *μ*M) and cisplatin (100 ng ml^−1^), the levels of p-ERK1/2 were decreased notably at 4 h (lanes 10 and 16; Figure 3A), 8 h (lanes 11 and 17; Figure 3A) and 24 h (lanes 12 and 18; Figure 3A) when compared to the control with the same dose of cisplatin and solvent (lanes 4–6; Figure 3A). These results indicated that U0126 successfully prevented the activation of ERK1/2 in the absence or presence of cisplatin.

We then studied the effect of U0126 on chemosensitivity to cisplatin in GH cells. We used MTT assay to determine the cell viability of GH cells in response to cisplatin for 4, 5, 6 days in the presence or absence of U0126 (20 *μ*M). As shown in Figure 3B–D, after exposure to five concentrations of cisplatin (1, 2, 3, 4 and 5 *μ*g ml^−1^), the percentage of cell viability in U0126-treated cells (filled columns) was higher compared with untreated cells (open columns) in a time-dependent manner. These results suggest that inhibition of MEK pathway is able to protect TGCT cells with high levels of MAD2 against cisplatin-induced cell death.

### Inactivation of MAD2 leads to downregulation of MEK/ERK pathway

**Figure 4:**
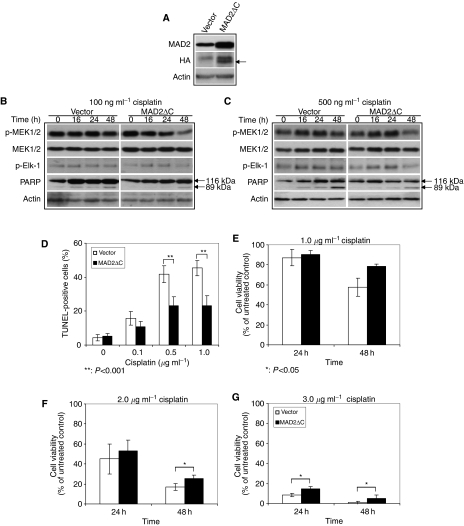
Effect of MAD2 inactivation on MEK pathway and cellular sensitivity to cisplatin in GH cells. GH cells were transiently transfected with a HA-tagged MAD2 dominant-negative construct (MAD2ΔC) and the expression of p-MEK and p-Elk, the percentage of TUNEL-positive cells and cell viability were examined after exposure to cisplatin. (**A**) MAD2 and HA protein expression in MAD2ΔC and vector transfected GH cells 48 h after transfection (arrow indicates the size of HA protein). (**B** and **C**) Expression of p-MEK1/2 and p-Elk-1 and PARP in GH cells expressing MAD2ΔC and the control vector after exposure to 100 ng ml^−1^ (**B**) and 500 ng ml^−1^ (**C**) cisplatin. Note that after exposure to cisplatin, the expression of p-MEK1/2 and p-Elk-1 was lower in the MAD2ΔC-transfected cells especially at 48 h post-exposure time compared to the vector control. (**D**) Terminal deoxynucleotidyl transferase-mediated nick end labelling staining of MAD2ΔC transfectants (filled columns) and control vector transfectants (open columns) after exposure to three doses of cisplatin for 4 days. Note that the percentage of TUNEL-positive cells in MAD2ΔC transfectants was lower than the control vector transfectants after cisplatin treatment. Results represented means of three independent experiments and error bars indicated standard deviation. (**E**–**G**) Cell viability of MAD2ΔC transfectants (filled columns) and control vector transfectants (open columns) after exposure to three concentrations of cisplatin for indicated time points. Note that the cell viability of MAD2ΔC transfectants was higher than that of control vector transfectants after treatment with same doses of cisplatin. Results represented means of three independent experiments and error bars indicated standard deviation.

To further investigate the correlation between MAD2-mediated cisplatin sensitivity and the MEK/ERK/Elk pathway, we inactivated MAD2 in GH cells by transient transfection of a HA-tagged dominant-negative construct of MAD2 (MAD2ΔC). GH cells were studied because of their relative high levels of MAD2 and high transfection efficiency. MAD2ΔC has been reported to override the function of MAD2, therefore, it is regarded as the dominant-negative form of MAD2 (Canman *et al*, 2002). As shown in Figure 4A, HA protein was detected in cells transfected with MAD2ΔC but not in the vector control cells (see the arrow). In addition, the MAD2 level was increased when compared with the vector control because the antibody used in this study was able to react with both endogenous MAD2 and ectopic HA-tagged MAD2ΔC proteins (Figure 4A). We also found that there was no significant difference in the basal level of p-MEK1/2 or p-Elk-1 between the MAD2ΔC transfectants and the vector controls (Figure 4B and C, 0 h time point), suggesting that MAD2ΔC alone did not alter the phosphorylation of these kinases.

Next, we determined whether inactivation of MAD2 could lead to any changes in the expression of MEK pathway-related proteins and cisplatin-induced apoptosis. After exposure to two concentrations of cisplatin (100 and 500 ng ml^−1^), the levels of p-MEK and p-ElK-1 were maintained at relatively high levels in the vector control but the levels of these proteins were decreased in a time-dependent manner in the MAD2ΔC transfectants (Figure 4B and C). We then evaluated if the decreased p-MEK1/2 and p-Elk-1 levels could lead to any changes in cisplatin-induced apoptosis by comparing the amount of cleaved PARP in the vector controls and MAD2ΔC transfectants after cisplatin treatment. In parallel with the decrease in p-MEK1/2 and p-Elk-1 protein levels, a reduction of cleaved PARP protein was also noted in the MAD2ΔC transfectants after cisplatin treatment. The relative amount of cleaved PARP was lower in the MAD2ΔC transfectants compared with the vector controls particularly at 48 h post-exposure time (Figure 4B and C; 100 and 500 ng ml^−1^). These results indicated that reduced levels of p-MEK1/2 and p-Elk-1 were associated with reduction of cisplatin-induced apoptosis in MAD2ΔC transfectants.

To further establish the association between MAD2 inactivation and cisplatin sensitivity, we performed TUNEL assay. As shown in Figure 4D, in contrast to the vector controls (open columns), the MAD2ΔC transfectants (filled columns) had significantly lower percentage of TUNEL-positive cells when the cells were exposed to the same doses of cisplatin (0.5 and 1.0 *μ*g ml^−1^ cisplatin, *P*<0.001). These results indicated that inactivation of MAD2 could lead to decreased cisplatin-induced apoptosis. 3-(4,5-Dimethyl thiazol-2-yl)-2,5-diphenyl tetrazolium bromide assay also confirmed these results that MAD2ΔC transfectants showed much higher cell viability (filled columns) compared with the vector controls (open columns) in response to same concentrations of cisplatin (Figure 4E–G). These results together with Western blot analysis (Figure 4B and C) indicated that inactivation of MAD2 led to the inhibition of MEK signalling pathway, which was associated with resistance to cisplatin-induced cell death in TGCT cells.

## DISCUSSION

In this study, we demonstrated that downregulation of MAD2 was associated with decreased sensitivity to cisplatin in non-isogenic TGCT cell lines. In addition, the positive association between MAD2 expression and cisplatin-induced MEK/ERK pathway activation indicates that the MEK/ERK signalling pathway may play an important role in the MAD2-mediated cisplatin sensitivity. Although previous studies have suggested a possible role of MAD2-mediated mitotic checkpoint in cellular response to DNA damage (Garber and Rine, 2002; Mikhailov *et al*, 2002), our results provide a possible molecular mechanism responsible for its action. However, the fact that a reverse correlation was observed between MAD2 expression and acquired cisplatin resistance in two pairs of TGCT cell lines suggests that MAD2 may play a different role in cisplatin sensitivity in the *in vitro* established acquired cisplatin-resistant TGCT cell lines.

MAD2 is localised to the kinetochore, a connection between the chromosome and the spindle, and it is required for generating the ‘wait’ signal in response to microtubule disruption. The MAD2-dependent spindle checkpoint acts to inhibit chromosome segregation until all chromosomes are properly attached to the spindle, ensuring accurate partition of the genetic material in order to preserve genome integrity (Rudner and Murray, 1996). Downregulation of MAD2 has been reported in several types of human cancer including lung (Takahashi *et al*, 1999), breast (Li and Benezra, 1996), nasopharyngeal (Wang *et al*, 2003) and ovarian carcinomas (Wang *et al*, 2002). Recently, several reports have suggested that extensive DNA damage is able to delay mitosis via activation of the MAD2-mediated spindle assembly checkpoint (Garber and Rine, 2002; Mikhailov *et al*, 2002; Nitta *et al*, 2004). For example, Garger and Rine (2002) indicate that spindle assembly checkpoint contributes to the cell cycle arrest to DNA-damaging agents in cells lacking either the DNA damage or the DNA replication checkpoints. In addition, it has been suggested that disruption of the function of kinetochore by the extensive DNA damage is able to delay mitotic exit by disruption of kinetochore functions but not by ATM-dependent mechanisms (Mikhailov *et al*, 2002). Recently, the toxicity of cisplatin has been correlated to its binding ability to tubulin, which leads to disruption of tubulin assembly and cell death (Tulub and Stefanov, 2001). These results support the hypothesis that mitotic checkpoint may play an important part in regulating cellular response to DNA damage. Previously, we have found that MAD2 overexpression leads to increased sensitivity to several DNA-damaging anticancer drugs, especially cisplatin, in NPC cells, which is associated with activation of the apoptosis pathway (Cheung *et al*, 2005). The results presented in the current study also showed that higher levels of MAD2 expression in TGCT cells were correlated with increased sensitivity to cisplatin (Figure 1, Table 2). In addition, inactivation of MAD2 by transfection of a dominant-negative MAD2 construct resulted in suppression of cisplatin-induced apoptosis and increased cell survival (Figure 4). These results indicate that it is possible that sufficient MAD2 expression in cancer cells may be able to facilitate the activation of the DNA damaged-induced apoptosis pathway, leading to chemosensitisation.

**Figure 5:**
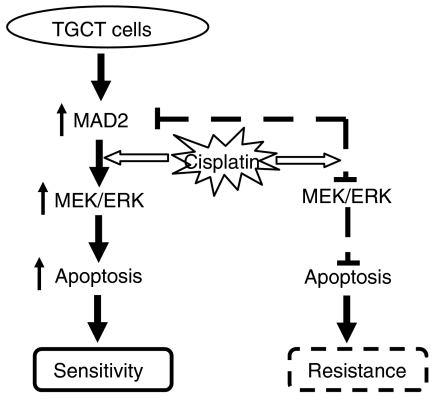
A schematic model of MAD2-mediated cisplatin sensitivity in TGCT cells.

The importance of MEK/ERK pathway in regulating apoptosis after cisplatin treatment has been suggested. For example, ERK activation is reported to be necessary for cisplatin-induced apoptosis in lung cancer cells (Wang *et al*, 2000), cervical carcinoma cell lines (Wang *et al*, 2000; Yeh *et al*, 2002) and malignant TGCT cell lines (Schweyer *et al*, 2004a). In addition, inactivation of MEK/ERK by its inhibitors has been shown to prevent apoptosis in response to cisplatin leading to resistance (Wang *et al*, 2000; Yeh *et al*, 2002), although contradictory results have been reported (Hayakawa *et al*, 1999; Persons *et al*, 1999). In this study, we found that activation of the MEK/ERK pathway was associated with increased sensitivity to cisplatin-induced apoptosis in the cells with high levels of MAD2 expression (Figure 2). Inactivation of the MEK kinase on the other hand, led to suppression of cisplatin-induced apoptosis in the sensitive cells expressing high levels of MAD2 (Figure 3). In addition, inactivation of MAD2 through transfection of a dominant-negative construct resulted in the suppression of cisplatin-induced MEK/ERK activation, reduction of cisplatin-induced apoptosis and cellular resistance (Figure 4). These results strongly suggest that the MAD2-mediated cisplatin sensitivity may be regulated through the MEK/ERK pathway. Although no direct link between MAD2 and the MEK/ERK pathway has been reported, several studies have indicated the importance of MEK/ERK pathway in regulating mitosis. For example, activation of the MEK/ERK pathway is required for exit from DNA damage-induced G2 cell cycle arrest (Abbott and Holt, 1999) and the transition from G2 into M (Lavoie *et al*, 1996; Wright *et al*, 1999). In addition, the localisation of active ERK to the mitotic kinetochore is also suggested to regulate proteins involved in chromosome segregation during metaphase to anaphase transition (Shapiro *et al*, 1998; Zecevic *et al*, 1998). Furthermore, ERK is also described to associate with kinetochores during early prophase, but this association is not apparent at later stages of mitosis (Knauf *et al*, 2005). The fact that MAD2 functions through its localisation to kinetochore suggests that there may be a direct interaction between the MEK/ERK pathway and MAD2 in regulating mitosis. Our results also agree with a study on one of the TGCT cell lines, NCCIT, that activation of MEK/ERK pathway is essential for the cisplatin-induced apoptosis which is associated with increased expression of several mitosis regulators such as cyclin B1 in response to cisplatin (Schweyer *et al*, 2004a). In addition, it is reported that *MAD2* mRNA levels are high in human testis and suggests that it may play a role in spermatogenesis (Pangilinan *et al*, 1997). It is possible that high levels of MAD2 expression in TGCT cells may function as a sensor for cellular damage (i.e. DNA damage) to facilitate activation of the DNA damaged-induced apoptosis through stimulation of the MEK/ERK pathway. This hypothesis may also explain the fact that why TGCT cells are hypersensitive to chemotherapeutic drugs, especially DNA-damaging agents such as cisplatin. Our data, in general, support the previous view of Schweyer *et al* (2004a, 2004b) that the cisplatin-induced apoptosis depends on MEK/ERK activation in TGCT cell lines. However, in the current study, in addition to confirming the importance of MEK/ERK pathway in cellular sensitivity to cisplatin, we have provided first link of this pathway to a key component of mitotic checkpoint control, MAD2, and our results suggest that the MAD2-mediated cisplatin sensitivity might be regulated through the MEK/ERK pathway (Figure 5).

The proposed MAD2-induced cisplatin sensitivity model (Figure 5) also helps to explain the relatively drug-resistant phenotype in yolk sac tumours. Histologically, GCTs with yolk sac tumour characteristics are often insensitive to cisplatin (Dunn *et al*, 1997), refractory to chemotherapy, relapse more frequently and associated with poor survival (Toner *et al*, 1991). In this study, the 1411HP cells that exhibit yolk sac tumour characteristics also had a relatively low MAD2 expression (Figure 1). In addition, there was a much lower p-MEK/ERK level after cisplatin treatment which was associated with decreased PARP cleavage (Figure 2A). It is possible that the decreased MAD2 expression in yolk sac tumour cells such as 1411HP may play a key role in suppression of cisplatin-induced MEK/ERK activation which leads to the suppression of apoptosis and resistance to cisplatin. Our findings are also supported by a previous report that cisplatin resistance in 1411HP cells was associated with higher threshold for apoptosis (Mueller *et al*, 2003).

In summary, we have demonstrated that high levels of MAD2 expression in TGCT cells are positively correlated with cellular sensitivity to cisplatin. In addition, the evidence that inactivation of MAD2 leads to suppression of MEK pathway in response to cisplatin suggests a novel underlying mechanism responsible for the MAD2-mediated cellular response to DNA damage. Furthermore, our results also implicate that downregulation of MAD2 may be an indicator for identification of cisplatin resistant TGCT patients. As cisplatin is one of the most widely use anticancer drugs, our findings may provide a novel therapeutic target for reversing cisplatin resistance through ectopic expression of the *MAD2* gene.

## Figures and Tables

**Table 1:** Characteristics of TGCT cell lines

**Cell line**	**Site of origin**	**Histology**	**Characteristics**	**References**
GH	Primary	NSGCT	EC	Wang *et al* (1997)
GCT27	Primary	NSGCT	EC	Pera *et al* (1987)
GCT27R^a^	Primary	NSGCT	EC	Kelland *et al* (1992)
NCCIT	Primary mediastinal	NSGCT	EC, S	Damjanov *et al* (1993)
NT2/D1	Lung metastasis	NSGCT	Pluripotent EC	Andrews *et al* (1984)
SuSa	Primary	NSGCT	EC, T	Hogan *et al* (1977)
SuSa-CP^b^	Primary	NSGCT	EC, T	Walker *et al* (1990)
833K	Abdominal metastasis	NSGCT	EC, T	Bronson *et al* (1980)
1411HP	Primary	NSGCT	EC, Y	Vogelzang *et al* (1985)
2102EP	Primary	NSGCT	EC	Wang *et al* (1980)

EC=embryonal carcinoma; NSGCT=non-seminomatous germ cell tumour; S=seminoma; T=teratoma; Y=yolk sac tumour.

a Acquired resistance cell line derived from GCT27.

b Acquired resistance cell line derived from SuSa.

**Table 2:** Summary of IC_50_ and IC_90_ doses of cisplatin and taxol in 10 TGCT cell lines

	**Cisplatin (ng ml^−1^)**		**Taxol (ng ml^−1^)**	
**Cell line**	**IC_50_±s.d.**	**IC_90_±s.d.**	**IC_50_±s.d.**	**IC_90_±s.d.**
GH	31.0±12.8	69.4±6.8	0.45±0.02	0.86±0.04
GCT27	69.2±10.4	162.1±18.3	0.82±0.02	1.66±0.11
GCT27R	242.8±8.5	665.6±21.4	0.71±0.01	1.29±0.12
NCCIT	38.80±5.0	99.6±15.1	0.85±0.08	1.67±0.29
NT2/D1	23.4±1.34	49.8±5.8	0.46±0.06	0.94±0.21
SuSa	17.1±0.4	57.3±3.4	0.62±0.01	1.05±0.04
SuSa-CP	139.0±13.1	345.5±29.5	0.65±0.02	1.29±0.12
833K	36.3±4.7	90.3±12.2	0.38±0.04	0.75±0.03
1411HP	189.4±21.9	471.7±41.9	0.75±0.03	1.50±0.22
2102EP	90.2±5.8	205.4±14.4	0.63±0.05	1.08±0.06
